# Low Vitamin D Levels in Children with Fractures: a Comparative Cohort Study

**DOI:** 10.1007/s11420-015-9447-7

**Published:** 2015-08-11

**Authors:** Peter D. Fabricant, Christopher J. Dy, Son H. McLaren, Ryan C. Rauck, Lisa S. Ipp, Shevaun M. Doyle

**Affiliations:** Department of Pediatric Orthopaedic Surgery, Hospital for Special Surgery, 535 East 70th Street, New York, NY 10021 USA; Washington University in St. Louis, 660 S. Euclid Ave, Box 8233, St. Louis, MO 63310 USA; New York Presbyterian Hospital/Weill Cornell Medical Center, 1300 York Ave, New York, NY 10065 USA; The Ohio State University College of Medicine, 338 W 10th Ave, Columbus, OH 43210 USA

**Keywords:** insufficiency, deficiency, calcium, fracture, BMI

## Abstract

**Background:**

The currently accepted ranges for “normal” serum vitamin D have recently been challenged in adults on the basis that healthy bone metabolism requires higher levels of vitamin D than previously thought.

**Purpose:**

The purpose of this study was to evaluate whether a new “biologically based” classification based on 25(OH)vitamin D levels that invoke an endocrine biomarker response (<20 ng/mL for deficiency and <32 ng/mL for insufficiency) is more appropriate for children with fractures than historical criteria.

**Methods:**

Serum 25(OH)vitamin D levels were collected from 58 children with acute low-energy fractures from an outpatient orthopedic clinic from 2009 to 2012. These vitamin D levels were compared with a cohort of 103 children with chronic kidney disease (CKD) from an adjacent clinic, a condition with acknowledged low levels of vitamin D. Then, the prevalence of vitamin D sufficiency in the fracture cohort was evaluated and compared using both historical guidelines and newer biologically based criteria.

**Results:**

25(OH)vitamin D levels in the fracture cohort did not differ from levels in the CKD cohort (27.5 vs. 24.6 ng/mL) indicating a similar distribution of vitamin D levels. This finding was consistent when controlling for significant covariables using linear regression analyses. In the fracture cohort, there was a discrepancy between historical and biologically based criteria in 64% of children.

**Conclusions:**

The results of the current study suggest that fracture patients are more frequently vitamin D deficient than previously thought. This finding is more readily apparent when newer biologically based criteria for vitamin D sufficiency are used.

**Electronic supplementary material:**

The online version of this article (doi:10.1007/s11420-015-9447-7) contains supplementary material, which is available to authorized users.

**Original Release Date: August 10, 2015**

**Expiration Date: August 10, 2016**

**Overview**

Pediatric fractures are common injuries and frequently seen during the course of normal development. The potential contribution of metabolic factors such as low serum vitamin D is receiving increasing attention. Understanding newer, biologically based criteria for vitamin D deficiency and insufficiency, as well as strategies for oral supplementation and interdisciplinary consultation with a pediatric endocrinologist, is critical when treating children with fractures.

**Learning Objectives**

Hospital for Special Surgery professional education activities are intended to improve knowledge, competence, and performance of our learners and to lead to better patient care. the conclusion of the activity, the participant should be able to:

- explain how to test for serum vitamin D levels and what thresholds denote vitamin D deficiency, insufficiency, and normal levels;
- identify how to test for vitamin D deficiency/insufficiency when clinically warranted in otherwise healthy children and to conduct appropriate vitamin D testing with pediatric patients in their practice;
- identify effective strategies for vitamin D supplementation based on measured serum vitamin D levels and to develop appropriate treatment plans for their patients including an oral supplementation strategy and a strategy for collaborating with a pediatric endocrinologist when needed.

**Target Audience**

This activity is targeted at orthopaedic surgeons, pediatricians, primary care physicians, rheumatologists, specialty physicians, physician assistants, residents, fellows and medical students.

**Accreditation**

Hospital for Special Surgery is accredited by the Accreditation Council for Continuing Medical Education to provide continuing medical education for physicians.

**Credit Designation**

Hospital for Special Surgery designates this Journal-based CME activity for a maximum of *1.0 AMA PRA Category 1 Credit(s)™*. Physicians should claim only the credit commensurate with the extent of their participation in the activity.

**Commercial Support**

This journal-based activity did not receive commercial support.

**Faculty Disclosure:**

In accordance with the Accreditation Council for Continuing Medical Education’s Standards for Commercial Support, all CME providers are required to disclose to the activity audience the relevant financial relationships of the planners, teachers, and authors involved in the development of CME content. An individual has a relevant financial relationship if he or she has a financial relationship in any amount occurring in the last 12 months with a commercial interest whose products or services are discussed in the CME activity content over which the individual has control.

It is the policy of Hospital for Special Surgery to disclose all financial relationships that planners, teachers, and authors have with commercial interests. Relationship information appears below:

**Activity Directors Disclosure:**

**Peter D. Fabricant, MD, MPH** has disclosed no relevant financial relationships.

**Shevaun M. Doyle, MD** has disclosed no relevant financial relationships.

**Planning Committee Disclosure:**

**Keren S. Baum, MA** has disclosed no relevant financial relationships.

**Charles N. Cornell, MD** has disclosed no relevant financial relationships.

**Shevaun M. Doyle, MD** has disclosed no relevant financial relationships.

**Christopher J. Dy, MD, MSPH** has disclosed no relevant financial relationships.

**Peter D. Fabricant, MD, MPH** has disclosed no relevant financial relationships.

**Natanya Gayle, MPH** has disclosed no relevant financial relationships.

**Lisa S. Ipp, MD** has disclosed no relevant financial relationships.

**Son H. McLaren, MD** has disclosed no relevant financial relationships.

**Ryan C. Rauck, BS** has disclosed no relevant financial relationships.

**OCME/CME Committee Disclosure:**

Hospital for Special Surgery Office of CME Staff and CME Committee members have no relevant financial relationships to disclose regarding this activity.

**Activity Faculty**

Activity Directors:

Shevaun M. Doyle, MD

Associate Attending Orthopaedic Surgeon

Hospital for Special Surgery

New York, NY

Associate Professor of Orthopaedic Surgery

Weill Cornell Medical College

New York, NY

Peter D. Fabricant, MD, MPH

Pediatric Orthopaedic Surgery Fellow

Children’s Hospital of Philadelphia

Philadelphia, PA

**Planning Committee**

Keren S. Baum, MA

Accreditation and Compliance Coordinator

Education & Academic Affairs

Hospital for Special Surgery

New York, NY

Charles N. Cornell, MD

Clinical Director of Orthopaedic Surgery

Attending Orthopaedic Surgeon

Hospital for Special Surgery

Professor of Clinical Orthopaedic Surgery

Weill Cornell Medical College

New York, NY

Shevaun M. Doyle, MD

Associate Attending Orthopaedic Surgeon

Hospital for Special Surgery

New York, NY

Associate Professor of Orthopaedic Surgery

Weill Cornell Medical College

New York, NY

Christopher J. Dy, MD, MSPH

Hand Surgery Fellow

Washington University Orthopedics

St. Louis, MO

Peter D. Fabricant, MD, MPH

Pediatric Orthopaedic Surgery Fellow

Children’s Hospital of Philadelphia

Philadelphia, PA

Natanya Gayle, MPH

Managing Editor, HSS Journal

Education & Academic Affairs

Hospital for Special Surgery

New York, NY

Lisa S. Ipp, MD

Associate Attending Pediatrician

Hospital for Special Surgery

Associate Professor of Clinical Pediatrics

Weill Cornell Medical College

New York, NY

Assistant Attending Pediatrician

New York-Presbyterian Hospital

New York, NY

Son H. McLaren, MD

Pediatric Resident

Weill Cornell Medical College

New York, NY

Ryan C. Rauck, BS

Medical Student

The Ohio State University College of Medicine

Columbus, OH

**TECHNICAL REQUIREMENTS**

**Windows:**

- Microsoft Windows XP, Windows Server 2003, Windows Vista, Windows Server 2008, Windows 7 operating system
- Microsoft Internet Explorer 6.0 SP1 or later, Firefox 2.0 or later, or Google Chrome 1.0 (Chrome is only supported on Mediasite version 5.0.3 and later) Web browser
- Windows Media Player 9 or later
- For Firefox and Chrome playback, Microsoft Silverlight 1.0 or later (viewers are prompted to install this plug-in when attempting to view a presentation)
- Broadband internet connection (256 Kbps or more).

**Mac – Requires Mediasite 4.3 and later:**

- Mac OS X 10.4.8 or later operating system
- Safari 2.0.4 or later or Firefox 2.0 or later Web browser
- Microsoft Silverlight 1.0 or later (viewers are prompted to install this plug-in when attempting to view a presentation).
- Broadband internet connection (256 Kbps or more).

For more information, please visit Sonic Foundry http://www.sonicfoundry.com/contact/.

Please view our privacy policy http://www.hss.edu/notice-of-privacy-practices.asp.

**Instructions for Posttest, Course Evaluation and CME Credit:**

In order to earn CME credit, you must complete an online posttest and evaluation following the completion of this activity. There is a passing requirement of 100%. Once you complete the posttest and subsequent evaluation, a certificate will be available for you to print.

For questions related to the posttest and subsequent evaluation, please contact HSS Journal at gaylen@hss.edu or 646-797-8509.

*Option 1: Take the posttest online.*

1. Go to the HSS Journal homepage at www.springer.com/hss.
2. Click on ‘CME and Free-to-Access Articles’ tab.
3. Click on “Low Vitamin D Levels in Children with Fractures: A Comparative Cohort Study ” to view the full-text pdf article.
4. After you have reviewed the article click on ‘Complete the Current CME Test Online’ to register and complete the test.

## Introduction

Pediatric fractures are common injuries [30] often sustained during the course of normal development, but the potential contribution of metabolic factors such as vitamin D insufficiency is receiving increasing attention [1, 5, 6, 8, 10, 12, 13, 16, 17, 22, 26, 36, 37, 39, 42, 45, 46, 50]. Historically, target serum vitamin D levels have been created using healthy population averages and standard deviations, and sufficiency thresholds were set at <11 ng/mL for deficiency, 11–20 ng/mL for insufficiency, and >20 ng/mL were considered normal. These thresholds were determined via an extensive systematic review of contemporary literature in American and Canadian populations by a task force from the Institute of Medicine (IOM) [3, 27, 41]. However, normal value ranges generated using this method are prone to error, as differences in sample demographics and disease prevalence between the population to be studied and the reference population may be present. This is particularly problematic with a condition that many consider under-recognized in the general population, such as vitamin D insufficiency.

Recognition of these limitations has prompted clinicians and researchers to consider newer evaluations of “normal” levels of vitamin D. Further, the measurement of serum biomarkers in adults has led to new knowledge that more vitamin D is required to suppress bone resorption than previously thought, shifting the “normal” range to higher values [25]. In vivo studies in adults have shown that 25(OH)vitamin D levels <30 ng/mL are associated with increased PTH [4]. In children, vitamin D is more critically involved with bone mineralization (rather than an osteopenic state), and it is therefore unclear if these more stringent biologically driven values for adults (<20 ng/mL, deficiency; 20–32 ng/mL, insufficiency; >32 ng/mL, normal) are applicable to children as well [50]. Currently, despite the frequency of fractures in children [30], there is a paucity of primary scientific literature that reports vitamin D levels in children with fractures. Understanding if otherwise healthy children with fractures have low vitamin D levels could prompt clinicians to screen for serum 25(OH)vitamin D levels at the first presentation of a fracture. In doing so, vitamin D supplementation could be added to the treatment plan in order to correct any abnormalities that would otherwise go unnoticed. While a recent meta-analysis has not supported the use of isolated vitamin D supplementation in adults to increase bone mineral density or reduce fracture risk, these results are not externally valid in children and adolescents, and preliminary data is needed to explore any associations between low vitamin D and fracture risk [47]. We hypothesized that 25(OH)vitamin D levels in pediatric fracture cohort would be low, and likely, no different than that of a comparison cohort of children with chronic kidney disease (CKD) well known to have low levels of vitamin D. We would also suggest that categorization of vitamin D sufficiency levels within the fracture cohort by historical methods are inadequate and should be replaced by a more stringent, biologically based categorization.

To that end, we designed and report herein an investigation comparing a cohort of children with nonoperatively managed low-energy long bone fractures who presented to an urban orthopedic specialty hospital with a comparison cohort of children with CKD from an adjacent affiliated academic referral center. Our primary research aim was to determine if the 25(OH)vitamin D levels in the fracture cohort would be different than that of a comparison cohort of children with CKD well known to have low levels of vitamin D. Our second aim was to calculate the rate of re-categorization of vitamin D sufficiency levels within the fracture cohort from historical methods to a more stringent, biologically based categorization.

## Patients and Methods

The study was approved by the hospital institutional review boards (IRB) at both involved institutions prior to the investigation and represents a retrospective analysis collected laboratory data. The vitamin D data acquired from the fracture cohort represents serum laboratory studies collected at the time of initial evaluation as part of the senior author’s (SMD) standard of care for children who sustain low-energy fractures. Inclusion criteria for the fracture cohort were patients seen in the pediatric (under 18 years old) fracture clinic from November 2009 (inception of routine 25(OH)vitamin D testing) through June 2012 with nonoperatively managed extraarticular fractures. Fracture pattern was determined by plain radiograph at the time of presentation by the senior author (SMD). Only low-energy fractures were included (e.g., ground-level falls or falls from less than 5 ft, fractures sustained during recreation/play, and not including fractures associated with motor vehicle injury). Exclusion criteria were any chronic illness other than seasonal allergies, any diagnosis of endocrine dysfunction, fractures of the small bones of the hands and feet (e.g., metacarpals, metatarsals, phalanges), and repeat fractures through a previously fractured site. This yielded a cohort of 58 otherwise healthy children who sustained low-energy fractures of the clavicle and long bones of the extremities. Serum levels of 25(OH)vitamin D and total serum calcium were collected at the first post-injury visit as part of the senior author’s routine practice. Additionally, patient demographic variables including age, gender, body mass index (BMI), race/ethnicity, and insurance payer status (government vs. private insurance) were recorded as part of standard office procedure.

Due to ethical and logistical institutional review board and clinical review panel restrictions regarding serum vitamin D testing on a control cohort of healthy children, a comparison cohort of children with CKD was drawn from an existing database. CKD was selected as a comparison cohort because of the acknowledged low levels of 25(OH)vitamin D associated with the condition [9, 19, 32, 34, 48, 51]. Inclusion criteria for the comparison cohort were patients seen in the adjacent pediatric nephrology clinic during the same time period (November 2009 though June 2012) with an active diagnosis of CKD and receiving their first serum 25(OH)vitamin D test. Subjects were excluded if they were already receiving vitamin D supplementation. This produced a comparison cohort of 103 non-supplemented children with a disease process known to have low levels of serum vitamin D. Available covariables collected in the institutional database included age, gender, BMI, race/ethnicity, insurance payer status (government vs. private insurance), and date of laboratory testing. Insurance payer status was included as a potential confounder of interest because of its known impact on access to musculoskeletal care for children and adolescents [15, 28, 29, 38, 44]. Because seasonal variation in sunlight exposure plays a critical role in cyclical vitamin D levels [20, 45], the distribution of seasonality of serum vitamin D testing was evaluated carefully for equality between groups using previously established methods [31, 39]. To that end, month and day of serum testing was classified based on season (December to March, winter; June to September, summer; other months, neither winter nor summer) and treated as a three-level discrete variable.

Data was collected using Microsoft Excel (Microsoft Corp., Redmond, WA, USA) and analyzed using SAS Software version 9.3 (SAS Institute, Inc., Cary, NC, USA). When the fracture cohort was compared to the CKD cohort, differences in serum vitamin D levels were evaluated using an independent sample *t* test after confirming data normality. Age, gender, race/ethnicity, BMI, insurance payer status (e.g., government insurance vs. private insurance), and seasonality of laboratory testing were all evaluated as potential confounding variables and controlled in linear regression analyses. All comparative analyses were two-tailed and used *p* = 0.05 as the threshold for statistical significance. All available patients meeting inclusion and exclusion criteria were used; therefore, an a priori power calculation was not performed [33]. Instead, a post hoc power calculation was performed using PS version 3.1.2 (Vanderbilt University, Nashville, TN, USA) to determine the threshold effect size that would be statistically significant between groups given the distribution of serum vitamin D levels in the fracture cohort.

In the subsequent evaluation of the fracture cohort in isolation, each child was classified into one of three categories (vitamin D deficient, vitamin D insufficient, and normal) for each of two classification schemata: “historical” (<11 ng/mL, deficiency; 11–20 ng/mL, insufficiency, >20 ng/mL, normal) [18, 40] and “biological” (<20 ng/mL, deficiency; 20–32 ng/mL, insufficiency; >32 ng/mL, normal) [11], based on previously described standards. Rates of category reclassification were recorded and analyzed using Fisher’s exact test.

## Results

No difference in serum vitamin D levels was detected between the fracture and CKD cohorts (Table 1). The fracture cohort was comprised of children with a variety of long bone fractures, with the most common being fractures of the distal radius (33%) (Table 2). No patient received supplementation with calcium or vitamin D outside of their normal dietary intake. There were no significant differences between the fracture and CKD cohorts in patient gender, BMI, or seasonality of vitamin D testing. There was no significant difference in vitamin D levels between those with government-issued insurance and private insurance. Differences in both age and race/ethnicity were noted between the fracture and CKD cohorts; the fracture cohort was younger (mean age 8.7 vs. 14.1, *p* = 0.01) and composed of more Caucasians (86 vs. 33%, *p* = 0.01). Raw mean vitamin D levels were not significantly different between cohorts, with a mean difference of 2.9 ng/mL (27.5 vs. 24.6 ng/mL, *p* = 0.18). Further linear regression modeling was performed to control for demographic variables that were noted to be different between cohorts (age, race/ethnicity), as well as those that have been shown previously to influence serum vitamin D levels (BMI [2, 17, 49, 52], seasonality of testing [20, 39, 45]), and continued to show no difference in serum vitamin D levels between cohorts (*p* = 0.71). A post hoc power analysis revealed that the study was powered to detect a difference in serum vitamin D of 4.5 ng/mL between groups, with an *α* = 0.05 and power = 0.8 [14].

**Table 1:** Serum vitamin D and demographic comparisons between the fracture cohort and the chronic kidney disease (CKD) cohort

Variable	Fracture cohort (*N* = 58)	CKD cohort (*N* = 103)	*p* value	Test
Serum 25(OH)vitamin D (ng/mL)	27.5 ± 9.6	24.6 ± 14.7	0.182	*t* test
Age (years)	8.7 ± 4.7	14.1 ± 5.2	<0.01*	*t* test
Gender (% male)	57%	52%	0.585	Chi-squared
Race/ethnicity (% Caucasian)	86%	33%	<0.01*	Chi-squared
Body mass index (kg/m^2^)	17.7 ± 3.1	18.4 ± 4.7	0.311	*t* test
Insurance payer status (% without private insurance)	19%	26%	0.299	Chi-squared

*CKD* chronic kidney disease

*p < 0.05

**Table 2:** Fracture characteristics of 58 children with acute low-energy long bone fractures

Fracture location	Number	Percent
Distal radius	19	33
Tibia	7	12
Clavicle	6	10
Supra/epicondylar humerus	6	10
Radius/ulna	6	10
Femur	4	7
Tibia/fibula	3	5
Ankle (distal fibula, extraarticular)	3	5
Radial neck	2	3
Proximal humerus	2	3

When the vitamin D level for each subject in the fracture cohort was evaluated with both “historical” and “biological” criteria, 64% of cases were reclassified, which was statistically significant (*p* < 0.001; Fisher’s exact test). Eighty-four percent (49/58) were “normal” using the historical classification, while only 34% (20/58) of the same patients remained “normal” after the application of a biologically based classification system (Table 3).

**Table 3:**
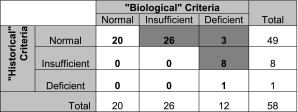
Sixty-four percent of children in the fracture cohort were reclassified to worse categories of 25(OH)vitamin D sufficiency when biologically based criteria were used, which was statistically significant (*p* < 0.001; Fisher’s exact test)

Gray shading indicates those children who were reclassified when biologically based criteria were implemented. “Historical Criteria”: <11 ng/mL, deficiency; 11–20 ng/mL, insufficiency; >20 ng/mL, normal. “Biological Criteria”: <20 ng/mL, deficiency; 20–32 ng/mL, insufficiency; >32 ng/mL, normal

## Discussion

In this comparative cohort study, we evaluated a cohort of children with nonoperatively managed low-energy long bone fractures and compared them with a cohort of children with chronic kidney disease (CKD). In doing so, the current study revealed that (1) children with fractures have low levels of serum vitamin D that were no different from a comparison cohort of children with CKD (a disease with known low serum 25(OH)vitamin D levels) with an acceptable amount of type II error; and (2) categorization of vitamin D sufficiency levels by historical methods may be inadequate in children with fractures as they resulted in a high rate of category reclassification when a more stringent (elevated) biologically based categorization was applied. Using the historical classification system in children with fractures would therefore lead to missed diagnoses of vitamin D insufficiency/deficiency.

Despite a comparative methodology designed to minimize bias, the current study has limitations. Due to ethical and logistical institutional restrictions, we were unable to collect serum vitamin D data from normal subjects and instead relied on an existing prospective database of laboratory results from patients with CKD. This is also a relative strength of the study, however, as comparison with a condition that is acknowledged to have low levels of 25(OH)vitamin D [9, 19, 32, 34, 48, 51] allowed us to establish that these otherwise healthy children with fractures are not “normal” with regard to serum vitamin D levels. Furthermore, the use of this database provided a comparison cohort and adequate power to detect a difference of 4.5 ng/mL in serum vitamin D concentration between the cohorts. Therefore, although we did not detect a difference in raw vitamin D levels between the fracture and CKD cohorts, our sample size provided an acceptable amount of type II error. It is important to also note that this study has shown that children with fractures have vitamin D levels that are no different than those of a CKD cohort known to have low levels of vitamin D; this should not be extrapolated to infer causative effect of decreased vitamin D on increased fracture risk. Strengths of this study are its use of prospectively collected data, the largest to date uniform sample of low-energy fractures in an otherwise healthy cohort of children, use of a comparison cohort, and the first report of vitamin D levels in children from metropolitan New York. While previous studies on vitamin D levels in children have been conducted in Boston (42° N) [20], Maine (44° N) [45], Georgia (33° N) [12], the UK (51° N) [10], Texas (29° N) [36], and New Mexico (31° N) [46], there are no reports of vitamin D levels in a pediatric patient population based in New York City (40° N), nor any data on a large cohort of children with various long bone fractures. One study, which recorded vitamin D levels in pediatric fracture patients, was done on 17 African-American children with forearm fractures in Washington DC [43]. However, this was a limited sample of 17 cases and provided only descriptive statistics without any comparative analysis.

In 2003, the American Academy of Pediatrics (AAP) [18] endorsed a recommendation made by the Institute of Medicine (IOM), which defined vitamin D deficiency as a serum vitamin D level below 11 ng/mL, insufficiency as levels between 11 and 20 ng/mL, and adequate vitamin D as a serum level above 20 ng/mL [27]. When the IOM updated the dietary reference intakes in 2011 [40], existing laboratory reference values were not addressed. The IOM [40] and Ross et al. [41] both highlighted the “urgent research and clinical priority” of reassessment of laboratory ranges for vitamin D. In adults, the historical level has since been considered too low, based on in vivo basic science research showing that vitamin D levels below 30 ng/mL are associated with increased PTH secretion [4, 11, 16]. This finding has led some to increase vitamin D insufficiency cutoff values in an effort to create a physiologically driven target serum concentration. The AAP recognizes these data on biomarkers (particularly PTH) in adults that have led to more stringent vitamin D target values; however, they remain undecided with regard to extrapolation of these data in children. The results of the current study suggest that vitamin D sufficiency thresholds in children should be revisited and reclassified based on this biomarker research.

This is the first study to evaluate vitamin D levels in a sample of children with acute low-energy fractures and compare them to a cohort of children with known low levels of vitamin D. The current study revealed that the serum 25(OH)vitamin D level in the fracture cohort was comparable to levels seen in a CKD cohort. This suggests that children with fractures may represent a population that is placed at a similarly increased risk for low serum levels of vitamin D, though the current study was not designed to establish population differences. While the absolute difference in serum 25(OH)vitamin D levels between cohorts was minimal (2.9 ng/mL), this small difference may even be an overestimate of the true difference between children with fractures and children with CKD in this study. Because it is known that non-Hispanic whites have higher serum concentrations of vitamin D than Blacks and Hispanics [23, 24, 35] primarily due to increased cutaneous synthesis of cholecalciferol [7, 21], the small difference in serum vitamin D concentration noted in the current study was likely partially driven by differences in race/ethnicity between the fracture and CKD cohorts. This remained statistically equivalent when controlling for race/ethnicity, in addition to age and BMI.

Despite this noted low serum concentration of vitamin D in the fracture cohort, when classified using historical criteria for vitamin D sufficiency, 80% of the fracture cohort had “normal” levels of serum 25(OH)vitamin D. Of this cohort, 64% were reclassified more appropriately to a lower category after the application of the newer biologically based criteria. This preliminary evidence indicates that the elevated target values for vitamin D more recently used in adults (<20 ng/mL for deficiency and <32 ng/mL for insufficiency) are more appropriate than historical pediatric standards in the setting of pediatric fractures.

Recommended dietary allowances of calcium and vitamin D were published in the 2011 report of the IOM and summarized by Ross et al. (Table 4). [41] In the senior author’s (SMD) practice, children with extremely low serum 25(OH)vitamin D (<12 ng/mL) are prescribed 5000 IU of vitamin D3 daily and referred to a pediatric endocrinologist immediately. Those with vitamin D insufficiency in the range of 12–20 ng/mL are treated with 5000 IU of vitamin D3 daily and rechecked in 3 months. Children with insufficiency in the 20–32 ng/mL range are treated with 2000 IU of vitamin D3 daily and rechecked in 6 months. If there is no improvement in vitamin D status at the time of repeat serum studies, serum biomarkers are obtained (PTH, ionized calcium, bone specific alkaline phosphatase, osteocalcin, and urine N-telopeptide), and the child is referred to a pediatric endocrinologist (Table 5).

**Table 4:** Pediatric Recommended Dietary Allowances (RDA) of calcium and vitamin D

Age	Calcium RDA^a^ (mg/day)	Vitamin D RDA^a^ (IU/day)
0 to 6 months old	200^b^	400^b^
6 to 12 months old	260^b^	400^b^
1 to 3 years old	700	600
4 to 8 years old	1000	600
9 to 18 years old	1300	600

Table adapted from data from Ross et al [41].

^a^Recommended Dietary Allowances (RDA) that meets the needs of ≥97.5% of population

^b^Represents Adequate Intake (AI) as RDAs were not established for infants

**Table 5:** Author’s preferred treatment algorithm for treating children with fractures and low serum 25(OH)vitamin D

Serum 25(OH)vitamin D level (ng/mL)	Amount of vitamin D3 supplementation (IU/day)	Interval prior to retest
<12	5000	Immediate referral to pediatric endocrinologist
12–20	5000	3 months
20–32	2000	6 months

Children who do not respond to supplementation are referred to pediatric endocrinology, and the following biomarkers are obtained: Serum PTH, ionized calcium, bone specific alkaline phosphatase, osteocalcin, and urine N-telopeptide

In conclusion, categorization of 25(OH)vitamin D sufficiency levels by historical methods may be inadequate in children with fractures, as they resulted in a high rate of category reclassification when a more stringent biologically based categorization was applied. Using the historical classification system could therefore lead to missed diagnoses of vitamin D insufficiency/deficiency, and in turn, a missed opportunity for treatment with oral supplementation. Like adults, the use of more stringent, biologically driven criteria for 25(OH)vitamin D sufficiency (<20 ng/mL for deficiency and <32 ng/mL for insufficiency) in children may be warranted. Future research should focus on determining if these results are generalizable to other pediatric disease states and on establishing more universal serum vitamin D screening protocols to optimize bone health in children with fractures.

## Electronic supplementary material

ESM 1 (PDF 1172 kb)
